# Lung Clearance Index and Quantitative Computed Tomography of Post-Infectious Bronchiolitis Obliterans in Infants

**DOI:** 10.1038/s41598-017-15330-8

**Published:** 2017-11-09

**Authors:** Yoon Hee Kim, Hyun Joo Shin, In Suk Sol, Soo Yeon Kim, Jong Deok Kim, Haesung Yoon, Kyung Won Kim, Myung-Joon Kim, Mi-Jung Lee, Myung Hyun Sohn

**Affiliations:** 1 Department of Pediatrics, Gangnam Severance Hospital, Seoul, South Korea; 2 Department of Radiology, Research Institute of Radiological Science, Severance Hospital, Seoul, South Korea; 3 Department of Pediatrics, Institute of Allergy, Brain Korea 21 PLUS Project for Medical Science, Severance Hospital, Seoul, South Korea; 4 Yonsei University College of Medicine, Seoul, Korea

## Abstract

Post-infectious bronchiolitis obliterans (BO) could be diagnosed via spirometry and chest computed tomography (CT); however, these tests are limited in infants. We aimed to evaluate the utility of lung clearance index (LCI) and air-trapping lung volume from chest CT in infants. This prospective study included 20 infants (mean age, 10.9 ± 6.3 months) diagnosed with post-infectious BO between 2009 and 2016. All subjects underwent multiple breath washout tests. For quantitative analysis of chest CT, the mean lung area attenuation value was used as an individual cutoff to determine the air-trapping lung volume. The mean cutoff lung attenuation value was −659 Hounsfield units, the mean total lung volume was 265 ml, and the mean air-trapping lung volume percentage was 22.9%. Functional residual capacity correlated with total lung volume and normal attenuation lung volume (*p* < 0.02). LCI (*p* < 0.02) and moment ratio (MR) 1 (*p* < 0.05) correlated with the air-trapping lung volume percentage. The concordance indices of LCI (0.659, *p* = 0.025) and MR1 (0.642, *p* = 0.046) were significantly correlated with the air-trapping lung volume percentage from CT. LCI and quantitative air-trapping lung volume from chest CT are feasible, complimentary tools for assessing infants with post-infectious BO.

## Introduction

Post-infectious bronchiolitis obliterans (BO) is the partial or complete obstruction of small airways by inflammation, granulation tissue, and/or fibrosis from recurrent pulmonary infection, especially during very early childhood (<6 months old)^1^. Recurrent epithelial injury in the bronchioles causes progressive narrowing, scarring, and chronic obstruction of the small airways^2^, resulting in multifocal air-trapping, bronchial wall thickening, bronchiectasis, and atelectasis, causing chronic severe symptoms in children^3^. A recent cohort study reported severely impaired pulmonary function among children with post-infectious BO after long-term follow-up^4^. Disproportional growth of lung parenchyma in airways of affected children was a proposed explanation for severe complication of post-infectious BO^5^. Therefore, early and accurate diagnosis of post-infectious BO in infants is critical to provide effective interventions and to predict disease outcome.

Diagnosis of post-infectious BO is usually based on clinical and radiological findings^4^. The spirometry aids the diagnosis of post-infectious BO in children, and it detects irreversible bronchiolar obstruction, and impaired lung distensibility^6^. However, the use of spirometry in infant with post-infectious BO is not feasible due to limited cooperation. Previous studies of spirometry in post-infectious BO usually focused on follow-up results^4,7,8^. Multiple breath washout (MBW) to calculate lung clearance index (LCI) has emerged as a sensitive marker to detect early small airway abnormalities; it has good reproducibility and accuracy in pediatric populations, including infants^9^. However, few studies have investigated LCI in post-infectious BO in infants, whereas several studies evaluated LCI in cystic fibrosis, asthma, and chronic lung disease in premature infants^10–13^.

Post-infectious BO can be diagnosed with high-resolution chest computed tomography (CT). The widely accepted radiological findings are bronchial wall thickening, bronchiectasis, segmental/subsegmental atelectasis, and mosaic attenuation patterns due to constrictive small airway obliteration^14–16^. CT is widely available in most institutes, and one study has demonstrated diagnostic value for gross CT findings in post-infectious BO^6^. Two studies attempted to quantify CT findings in children with BO^17,18^. However, to our knowledge, no previous study has quantitatively analyzed air-trapping lung area in chest CT in infants with post-infectious BO.

This study had two objectives: (1) to determine the usefulness of LCI from MBW and quantification of air-trapping lung volume from chest CT for assessing the degree of small airway obstruction in infants with post-infectious BO, and (2) to identify existing correlations between LCI and air-trapping lung volume measured in chest CT scans of infants.

## Results

### Subject Characteristics, Quantitative CT Assessment, and Infant PFT

The clinical characteristics of all 20 infants diagnosed with post-infectious BO in this study are presented in Tables 1 and 2. The mean age at the time of diagnosis was 10.9 months, and 18 infants (90%) were male. The mean percentiles of height and weight were 56.6 and 52.2, respectively. The pathogens involved in the initial lower respiratory infection that might cause post-infectious BO included adenovirus, parainfluenza, and *Mycoplasma pneumoniae*. The mean duration of recurrent and persistent airway obstruction symptoms were 3 months, and the mean interval period between infant PFT and chest CT was 13.5 days.

**Table 1:** Subject Characteristics and Quantitative Computed Tomography Assessment.

	Subjects (*n* = 20)
**Age at diagnosis, month**	10.9 ± 6.3
**Male gender, no (%)**	18 (90)
**Causative pathogen of initial lower respiratory infection, no (%)**	
Adenovirus	2 (10)
Influenza	1 (5)
Parainfluenza	4 (20)
Respiratory syncytial virus	2 (10)
Metapneumovirus	2 (10)
*Mycoplasma pneumoniae*	3 (15)
Other	3 (15)
Unknown	3 (15)
**Duration of recurrent airway obstruction symptoms, month**	3.0 (2.0−3.8)
**Interval between infant PFT and CT, day**	13.5 (2.8−54.0)
**Quantitative CT**	
**Measured attenuation**	
Normal lung area attenuation (HUn), HU	−560 ± 68
Air-trapping lung area attenuation (HUa), HU	−759 ± 60
Cutoff lung attenuation (HUc), HU	−659 ± 60
**Calculated lung volume**	
Total lung volume, ml	265 ± 127
Normal attenuation lung volume, ml	191 ± 72
Air-trapping lung volume, ml	43 (11−120)
Air-trapping lung volume percentage, %	22.9 ± 18

Data are expressed as number (percentage), mean ± standard deviation, or median (interquartile range).

PFT, infant pulmonary function test; CT, computed tomography; HU, Hounsfield units.

**Table 2:** Infant Pulmonary Function Tests with Bronchodilator.

	**Subjects (** ***n*** ** = 19)**		
**Age at infant PFT, month**			
**Height, cm**	76.8 ± 9.4		
Height percentile for same age and gender	56.6 ± 33.5		
**Weight, kg**	9.7 ± 2.4		
Weight percentile for same age and gender	52.2 ± 33.3		
**Infant PFT**	**Before BD treatment**	**After BD treatment**	***p***
**Tidal breathing**			
Tidal volume, ml	77.8 ± 35.1	83.8 ± 36.1	0.172
Respiratory rate breaths per min	34.5 ± 6.3	34.2 ± 6.1	0.625
Tidal breathing ratio (t_PTEF_/t_E_)	29.3 ± 13.2	24.5 ± 7.1	0.042
**Multiple breath washout**			
Functional residual capacity (FRC), ml	167 ± 52	151 ± 39	0.014
Lung clearance index (LCI)	8.38 ± 1.81	8.88 ± 1.71	0.225
Moment ratio 1 (M1/M0)	2.65 ± 0.68	2.80 ± 0.61	0.241
Moment ratio 2 (M2/M0)	13.83 ± 8.25	15.15 ± 7.52	0.365

Data are expressed as number (percentage), mean ± standard deviation, or median (interquartile range).

BD, bronchodilator; PFT, pulmonary function test; *p*, paired *t*-test.

**Figure 1:**
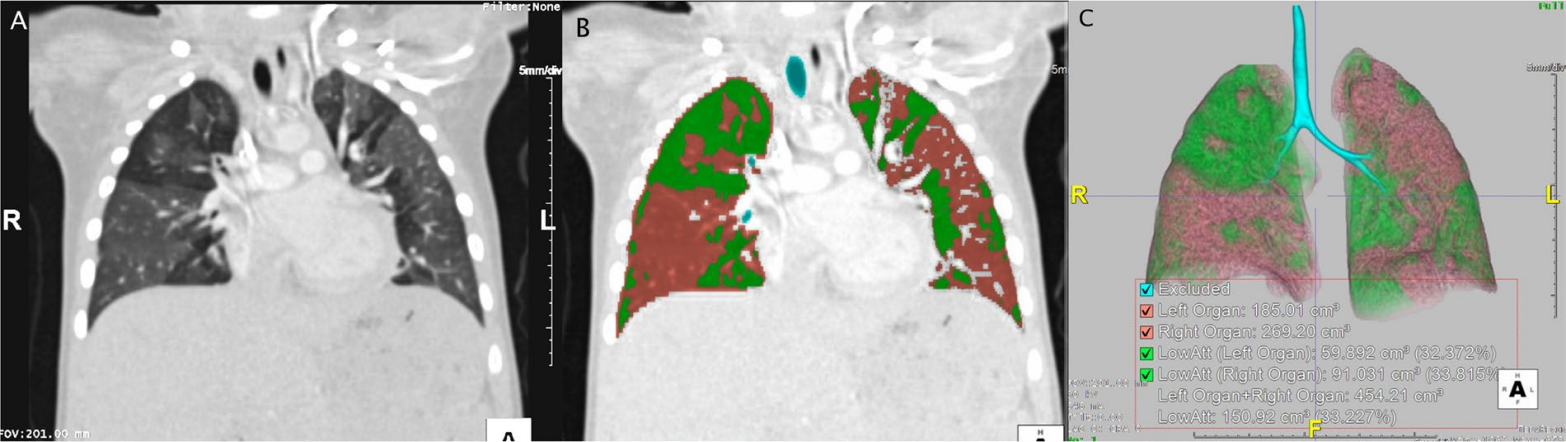
Quantitative chest CT assessment in a 21-month-old boy with post-infectious bronchiolitis obliterans. (**A**) Coronal image shows differing multifocal attenuation areas in both lungs from air-trapping and normal aeration. Normal lung attenuation value (HUn) was -568.4 HU and air-trapping lung attenuation (HUa) was -836.8 HU in this patient. (**B**) Both lungs were segmented computationally by applying the cutoff attenuation value (HUc, -702.6 HU for this patient). The same coronal image in part (**A**) shows the air-trapping areas in green, the normally aerated lung in orange, and the excluded airway in blue. (**C**) The 3D color image shows the calculated total lung volume (454 ml), air-trapping lung volume (151 ml), and air-trapping lung volume percentage (33.2%) in this patient.

The results of quantitative CT assessment are presented in Fig. 1 and Table 1. The mean attenuation of normal lung area (HUn) was −560 HU, and that of air-trapping lung area (HUa) was −759 HU. The mean cutoff lung attenuation (HUc) was −659 HU. The mean calculated total lung volume was 265 ml, and the mean percentage of air-trapping lung volume was 22.9%.

The results of infant PFT with bronchodilator treatment are presented in Table 2. The mean LCI values before and after bronchodilator were 8.38 and 8.88, respectively. The tidal breathing ratio (t_PTEF_/t_E_) and FRC differed before and after bronchodilator treatment, whereas LCI, MR1, and MR2 did not differ.

### Quantitative CT Assessment and Infant PFT Parameters

**Figure 2:**
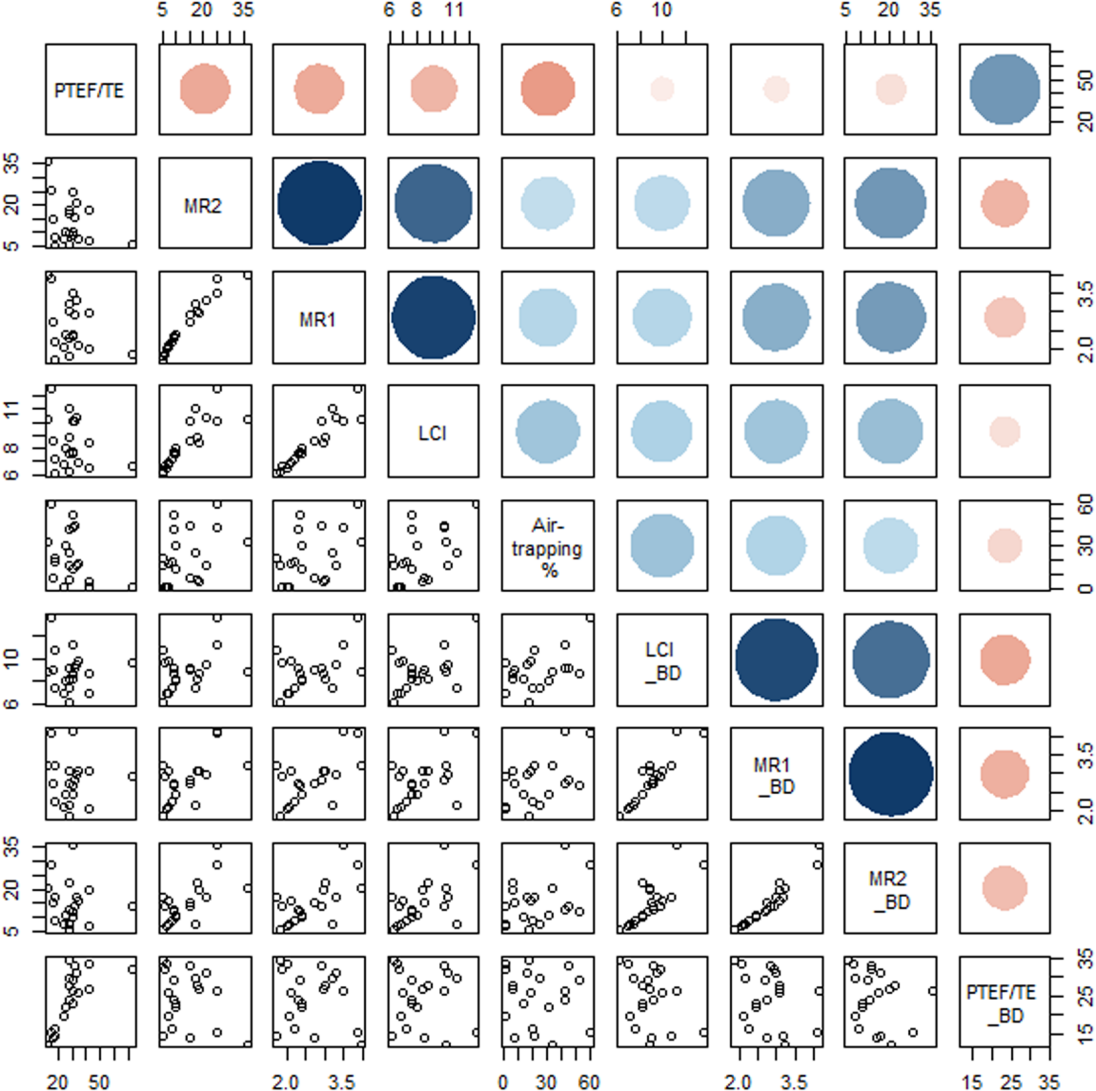
Scatter plot matrix illustrates the general correlation among air-trapping area percentage of chest CT and infant pulmonary function test parameters representing small airway obstruction, lung clearance index (LCI), moment ratio (MR)1, MR2, and tidal breathing ratio (t_PTEF_/t_E_) before and after bronchodilator (BD) treatment. The variables are written in a diagonal line from top left to bottom right. Each variable is plotted against all others in the left lower triangle, and the correlation significance is shown as a circle (blue circle, positive correlation; red circle, negative correlation).

LCI, MR1, MR2, and tidal breathing ratio were possible indices of small airway obstruction. Therefore, general correlations between these infant PFT parameters and the air-trapping lung volume percentage of the quantitative CT were analyzed in a correlation matrix (Fig. 2). The tidal breathing ratio (t_PTEF_/t_E_) did not tend to correlate with air-trapping lung volume percentage, whereas the other parameters before bronchodilator treatment tended to correlate with air-trapping lung volume percentage. All MBW parameters, LCI, MR1, and MR2, were well-correlated with each other.

**Figure 3:**
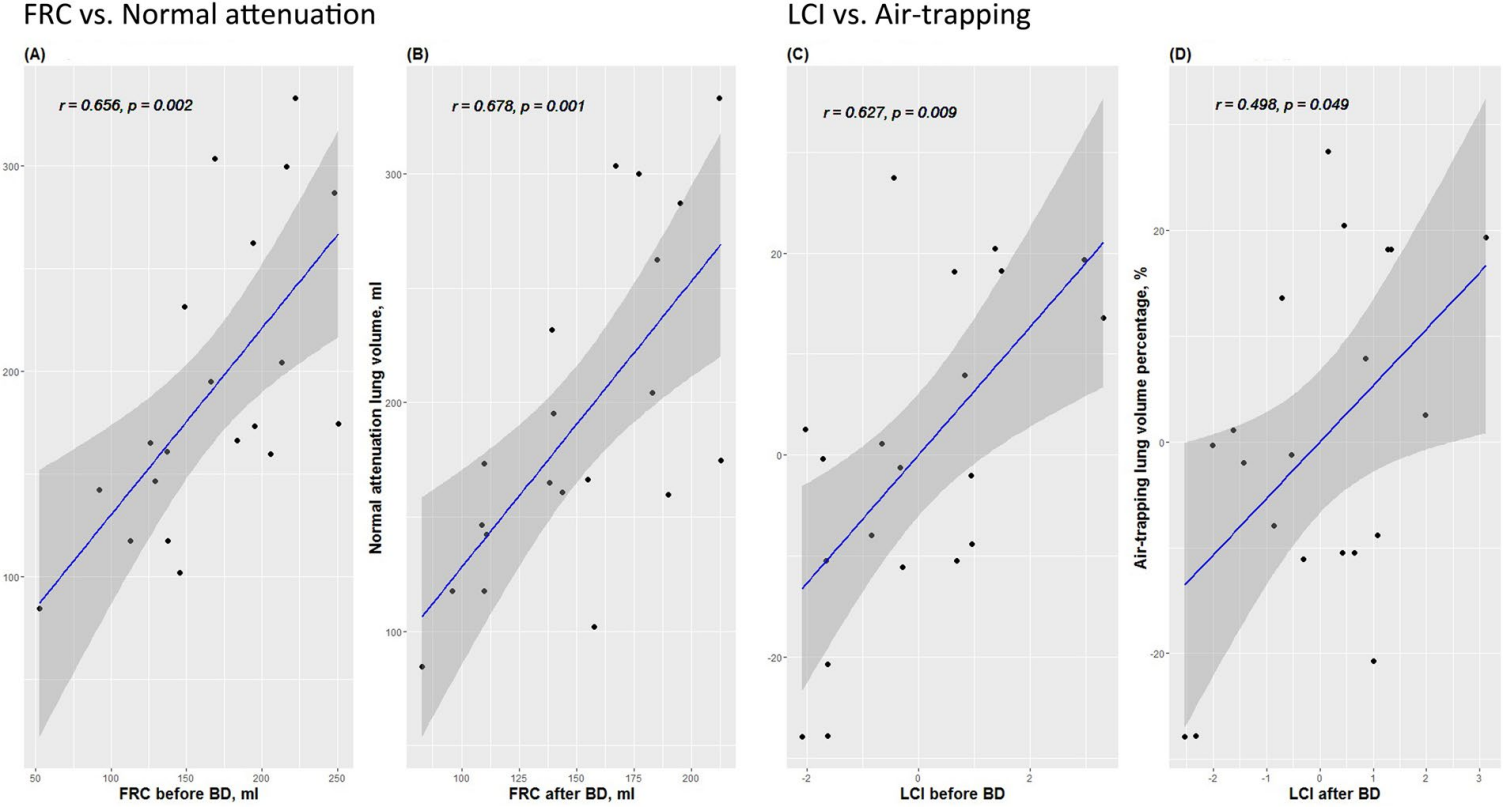
Scatter plots for correlation analyses of the quantitative chest CT assessment and multiple breath washout (MBW) parameters. Normal attenuation lung volume and functional residual volume (FRC) (**A**) before bronchodilator (BD) treatment, and (**B**) after BD treatment. Air-trapping lung volume percentage and lung clearance index (LCI) (**C**) before BD treatment, and (**D**) after BD treatment.

FRC values before and after bronchodilator were well-correlated with total lung volume and normal attenuation lung volume of the quantitative CT assessment (Table 3 and Fig. 3). The correlation matrix analysis revealed that LCI, MR1, and MR2 seemed to be related to the air-trapping lung volume percentage. Thus, partial correlation analysis was conducted on these data using additional models that adjusted for confounding factors. Infant age, gender, height, and weight were adjusted in model 2. In model 3, these four factors, as well as the interval between PFT and CT scan, were adjusted. LCI and MR1 before bronchodilator treatment were well-correlated with air-trapping lung volume percentage in all models. LCI and MR1 after bronchodilator were correlated with air-trapping lung volume percentage only in model 1, and these correlations were not preserved after adjusting for the confounding factors considered in models 2 and 3. MR2 was not significantly correlated with air-trapping lung volume percentage in any model.

**Table 3:** Correlation Analysis Between MBW Parameters and Quantitative Computed Tomography Assessment Parameters.

**Quantitative CT parameters**		**FRC before BD treatment**		**FRC after BD treatment**	
		***r (p)***		***r (p)***	
Total lung volume, ml		0.518 (0.019)		0.533 (0.015)	
Normal attenuation lung volume, ml		0.656 (0.002)		0.678 (0.001)	
		**LCI before BD treatment**		**LCI after BD treatment**	
		***r (p)***		***r (p)***	
Air-trapping lung volume percentage, %	Model 1	0.541 (0.014)		0.550 (0.012)	
	Model 2	0.627 (0.009)		0.498 (0.049)	
	Model 3	0.608 (0.016)		0.465 (0.081)	
		**Before BD treatment**, ***r (p)***		**After BD treatment**, ***r (p)***	
		**MR1**	**MR2**	**MR1**	**MR2**
	Model 1	0.456 (0.044)	0.377 (0.101)	0.483 (0.031)	0.404 (0.077)
	Model 2	0.519 (0.039)	0.400 (0.125)	0.477 (0.061)	0.439 (0.089)
	Model 3	0.518 (0.048)	0.429 (0.111)	0.448 (0.094)	0.406 (0.133)

Data are expressed as *r* (correlation coefficient) and *p* value adjusting for age, gender, height, and weight in model 2, and adjusting additionally for interval between infant PFT and CT in model 3.

BD, bronchodilator; MBW, multiple breath washout; CT, computed tomography; FRC, functional residual capacity; LCI, lung clearance index; MR, moment ratio; PFT, pulmonary function test.

### Concordance between Quantitative CT Assessment and Infant PFT Parameters

The concordance index between air-trapping lung volume percentage and MBW parameters are presented in Table 4. The concordance index of LCI and MR1 before bronchodilator was significant (0.659 with *p* = 0.025 for LCI; 0.642 with *p* = 0.046 for MR1). No other values had significant concordance indices with respect to air-trapping lung volume percentage.

**Table 4:** Concordance Analysis Between Lung Clearance Index and Air-Trapping Lung Volume Percentage.

Air-trapping lung volume percentage, %		Concordance index (SE)	95% CI	*p*
LCI	Before BD treatment	0.659 (0.071)	0.518, 0.795	0.025
	After BD treatment	0.648 (0.076)	0.500, 0.789	0.051
MR1	Before BD treatment	0.642 (0.071)	0.503, 0.776	0.046
	After BD treatment	0.624 (0.074)	0.479, 0.758	0.094
MR2	Before BD treatment	0.620 (0.073)	0.474, 0.753	0.100
	After BD treatment	0.607 (0.074)	0.468, 0.747	0.148

Data are expressed as concordance index (SE).

BD, bronchodilator; LCI, lung clearance index; MR, moment ratio; SE, standard error; CI, confidence interval.

^*^ *P* was determined using bootstrapping methods.

## Discussion

Our study demonstrates the usefulness of MBW for calculating FRC and LCI and chest CT for quantifying the air-trapping lung volume; these parameters enable assessment of small airway obstruction in infants with post-infectious BO. Normal and air-trapping lung areas on CT images were differentiated using the mean lung area attenuation value as a cutoff attenuation value (HUc). The calculated air-trapping lung area showed good correlation and concordance with LCI, implicating airway obstruction in infants with post-infectious BO.

Post-infectious BO frequently affects infants younger than 6 months of age. It causes severe, irreversible, and persistent small airway obstruction and impairs pulmonary function as the child develops^19^. Several studies have used serial spirometry to demonstrate functional impairment in children afflicted with post-infectious BO^2,4,5^. However, data are lacking regarding the initial diagnosis of post-infectious BO using PFT in infants. Few studies have investigated computational quantification of air-trapping lung volume in infant chest CT images for post-infectious BO either.

Spirometry and chest CT are considered to be the most useful objective assessment tools for chronic lung disease in infants and children. However, these tools are not sensitive enough to detect early airway changes^20^. Several recent studies of cystic fibrosis in children suggested the usefulness of LCI for assessing early stages of chronic lung disease, and demonstrated good correlation with structural lung abnormalities in chest CT^10,11,21,22^. This study showed that LCI also was a good index for evaluating lung abnormalities in infants with post-infectious BO, another chronic lung disease that results in severe insult to the lower respiratory tract^19^.

CT is usually more valuable than spirometry to diagnose post-infectious BO because CT findings, such as mosaic attenuation, can be more specific than spirometry findings, such as fixed airway obstruction^22,23^. To assess treatment response or follow-up, spirometry is usually preferred over CT because it reflects clinical symptoms and performance. Moreover, CT images are difficult to quantitate and repeated CT scans are potentially harmful due to cumulative radiation exposure^22–24^. However, because spirometry in infants or young children is difficult due to limited cooperation, there is no choice but to use CT to assess follow-up^3,25^. Our study is meaningful because it not only suggests that CT quantification is feasible and reliable, but also that the MBW test is useful to assess infant pulmonary function in post-infectious BO.

All subjects in this study had good prognoses within the spectrum of post-infectious BO because they were discharged from the hospital without any oxygen or mechanical ventilation therapy or morbidity. This study showed that LCI and quantification of air-trapping lung areas from chest CT was feasible for post-infectious BO in infants; therefore, the usefulness of these two tools could be tested for assessing the early stage lung abnormalities. The LCI values before and after bronchodilator treatment were 8.38 and 8.88, respectively, which were above the cutoff values of approximately 7.0 to 7.5 suggested in other studies, indicating that LCI could be used as an independent marker for evaluating lung abnormality in post-infectious BO^11,21,26^. Future studies that include a healthy control population and many subjects with post-infectious BO are needed to determine a reliable cutoff value of LCI.

Although the MR could be more sensitive than LCI for detecting the early lung abnormalities, it is not yet acceptable in clinical relevance and various researches^11,27^. Here, MR1 showed good correlation with air-trapping lung volume from the chest CT scan, possibly because MR2, which reflected the latter portion of the washout curve, could be unstable in a small unhealthy infant who could have some difficulty performing the MBW test, especially during sedation. For this reason, different cutoffs of LCI and MR were suggested for better diagnostic performance according to the different final gas concentration^26,28^. Here, the tidal breathing ratio (t_PTEF_/t_E_) was not correlated with the air-trapping lung volume from chest CT or with the MBW parameters (LCI and MR). These results were consistent with those from studies on infant cystic fibrosis^29^.

There has been some controversy regarding bronchodilator response in post-infectious BO, although fixed irreversible airway obstruction is widely accepted^1^. A subsequent treatment involving an inhaled corticosteroid and a bronchodilator is recommended in cases where the patient experiences improvement in airway obstruction after a bronchodilator treatment^1^. All subjects in our study inhaled a short-acting bronchodilator during hospitalization. We could not control for inhaled bronchodilator use before chest CT; therefore, we analyzed infant PFT parameters before and after bronchodilator. The good correlation of LCI and MR1 with air-trapping lung volume from CT was preserved independent of bronchodilator use.

Several studies have attempted to define and quantify chest CT findings for the assessment of small airway disease. Direct quantification of small airways (<2 mm in diameter) is not possible using CT scans because the resolution of the scan does not permit it. Therefore, quantification of air-trapping area on expiratory phase CT has been evaluated to assess small airway disease^30^. Previous studies in adults used threshold values of -856 or -950 HU for quantification of air-trapping areas in chronic obstructive pulmonary disease or emphysema, and recent studies used attenuation differences between inspiration and expiration CT images to determine accurate threshold attenuation values^30–32^.

The method used to define the cutoff value between normal and air-trapping lung areas in CT has not yet been standardized in children. The normal lung attenuation value is higher in children than in adults, and increases in younger children^33^. A previous study reported that the lung attenuation value in neonates was -380 HU^33^. Therefore, the adult threshold value is not suitable for children and infants. The first attempt to quantify the air-trapping lung area in pediatric chest CT was reported by Kim *et al*.^18^. They calculated individualized threshold values using mean lung area attenuation values in children with BO from graft-versus-host disease (GVHD), rather than using fixed threshold values. Here, we used this same method, and demonstrated the feasibility of this method in infants with post-infectious BO. The quantified lung volumes in CT were significantly correlated with FRC and LCI.

We also demonstrated the usefulness of advanced computational tools that can perform segmentation according to lung parenchymal attenuation. It is challenging to differentiate air-trapping areas according to the HU values in routine chest CT in infants, because deep inspiration images cannot be obtained, respiratory rates are normally faster in infants, and the image contrast from soft tissue is limited in the small thoracic cage of infants. Most studies use qualitative or semi-quantitative methods, such as scoring systems for pediatric chest CT^11,34,35^. A few quantitative methods have been applied for children > 2 years old with cystic fibrosis or GVHD-related BO^18,36,37^. One study calculated airway and lung volumes for infants with chronic lung disease, and another study used xenon-ventilated dual-energy CT to differentiate air-trapping areas in pediatric lungs^17,38^. Our study was the first attempt to demonstrate the utility of routine chest CT for quantitative assessment of air-trapping lung volume and lung function in infants. Chest CT with variable breath phases could be used to quantify lung volumes with different attenuations, and this comparative method, like PFT, can be used to evaluate obstructive airway disease.

The study has several limitations and strengths. First, the study size was small because few infants diagnosed with post-infectious BO underwent both infant PFT and chest CT. Despite its size, this study is meaningful because it represents the first attempt to use both initial PFT and quantitative air-trapping lung volume analysis of chest CT in infants with post-infectious BO, and the results indicate clinical utility. To overcome statistical limitations from study size, we conducted concordance analysis additionally using matching orders of two variables. Second, the measured amount of air-trapping lung volume can be affected by different breathing phases, different CT machines, and different CT parameters. We could not control specific breathing maneuvers in infants under shallow sedation without intubation or ventilation. The correlation coefficient or *p* value would likely be more significant if the air-trapping lung volume in full expiration phase could be obtained. This was a long-term study (8 years), and thus the CT machines and parameters in use changed with time. However, the results found here provide rationale for future studies using the same CT protocol and respiratory phase.

This study clarified that the initial LCI and method to quantify air-trapping lung volume from chest CT are feasible and useful for assessing obstructive small airway disease in infants with post-infectious BO. The use of mean lung area attenuation value as a cutoff attenuation value was appropriate to quantify normal and air-trapping lung areas in infant chest CT. Initial LCI and air-trapping lung volume percentage in chest CT showed good correlation and concordance. These could provide supplementary tools for diagnosis and follow-up of lung function in infants with post-infectious BO.

## Methods

### Study Design and Subjects

Infants diagnosed with post-infectious BO between 2009 and 2016 at the Severance Children’s Hospital were enrolled. All subjects were < 2 years old at the time of initial diagnosis. The post-infectious BO diagnosis was based on the following: (1) history of lower respiratory infection before at least 60 days; (2) persistent or recurrent airway obstruction symptoms characterized by tachypnea, increased anteroposterior chest diameter, crackles, wheezing, and hypoxemia for at least 60 days; (3) typical chest CT images such as mosaic attenuation, air-trapping, bronchial wall thickening, bronchiectasis, and/or segmental/subsegmental atelectasis^14–16^. Patients were excluded if they had other chronic lung diseases (e.g., asthma, bronchopulmonary dysplasia, aspiration pneumonia) or other conditions that could affect lung function (e.g., congenital heart disease, neuromuscular disease, severe developmental delay, prematurity less than 34 weeks’ gestation).

After initial diagnosis, all infants underwent infant pulmonary function test (PFT) at the next visit to the out-patient clinic, and were asked to interrupt their inhaled or oral corticosteroid and/or β2-agonist for at least 24 h before the infant PFT. None of the subjects had symptoms of acute respiratory infection, such as fever or acute exacerbation, for at least 1 week before the infant PFT.

This study was approved by the Institutional Review Board of Severance Hospital (Protocol no. 4-2007-0255) and all methods in this study were performed in accordance with the relevant guidelines and regulations of this ethics commitee. Written informed consent was obtained from the parents.

### Quantification of Air-Trapping in Chest CT

All infants underwent CT scans of the chest and details regarding the methods of CT scan are provided in an online data supplement.

One of two experienced pediatric radiologists quantified normal and air-trapping lung areas (Fig. 1). The radiologist reviewed chest CT images in soft kernel with a lung window setting of 1500/−500 Hounsfield units (HU) using a picture archiving and communication system (PACS) workstation (Centricity Radiology RA1000; GE Medical Systems, Milwaukee, WI). Using the most representative axial image that clearly depicted both normal and air-trapping lung areas without artifact, two circular regions of interest (ROIs) with 2−3 mm diameter were drawn in each area, avoiding pulmonary vessel, airways, or dependent portions of both lungs to minimize the effect of dependent atelectasis in infants. The average attenuation values of normal (HUn) and air-trapping (HUa) lung areas within the ROIs were automatically computed based on the delineated ROIs. Then, the cutoff attenuation (HUc) was calculated as the mean lung area attenuation value using the following equation: HUc = (HUn + HUa)/2. The HUn, HUa, and HUc values were recorded for each patient.

Lung parenchyma was segmented computationally by subtracting chest wall, mediastinum, and grossly visible major airways from CT images using Aquarius iNtuition Viewer (version 4.4.12, TeraRecon, San Mateo, CA, USA). Next, the air-trapping and normal lung areas were segmented in both lungs according to the calculated HUc value. The normal lung attenuation was defined as the area with a higher attenuation value than the HUc, whereas air-trapping lung was defined as the area with a lower attenuation value than the HUc. The volume of each area was automatically computed. We also did visual checks to make sure that the trapped air detection reasonably approximated the visual appearance. Total lung volume, normal attenuation lung volume, and air-trapping lung volume were recorded. The percentage of air-trapping lung volume was calculated by dividing the air-trapping lung volume by the total lung volume.

### Infant Pulmonary Function Tests

Multiple breath washout (MBW) tests were conducted using the EXHALYZER D system (Eco Medics AG, Durnten, Switzerland) with 4% sulfur hexafluoride (SF_6_). Subjects were sedated with orally administered chloral hydrate (60−100 mg/kg). The PFT was conducted in the supine position, with the infant’s head in the midline using a size 0 round infant silicone face mask placed over the nose and mouth. Details regarding the methodology used for performing of infant PFT are provided in an online data supplement.

Reversibility testing with a bronchodilator was conducted for tidal breathing flow-volume loops (TBFVL) and MBW tests. Salbutamol (2.5 mg/2.5 ml) was administered with a Pari LC-Jet Plus nebulizer and a face mask (Pari Respiratory Equipment, Starnberg, Germany). TBFVL and MBW tests were conducted before the administration of nebulized salbutamol and 15 min after administration.

The following infant PFT data were derived from the tidal breathing test: tidal volume, respiratory rate breaths per min, and tidal breathing ratio (time to peak expiratory flow/expiratory time, t_PTEF_/t_E_). Functional residual capacity (FRC), LCI, moment ratio 1 (MR1; M_1_/M_0_), and moment ratio 2 (MR2; M_2_/M_0_) were derived from the MBW test.

### Statistical Analysis

Reported values in the text and tables are means ± standard deviation, median values with interquartile ranges, or number and percentage, as appropriate. Differences in PFT parameters obtained before and after bronchodilator were analyzed by the paired *t*-test. Correlation matrix, Pearson’s correlation, and partial correlation analyses were used to assess the relationship between PFT parameters and quantitative CT assessment parameters. FRC was compared with total lung volume and normal attenuation lung volume on CT using Pearson’s correlation analysis. LCI was compared with air-trapping lung volume percentage using Pearson’s and partial correlation analyses, adjusting for age, gender, height, and weight in model 2 and additionally adjusting for interval between PFT and CT in model 3. The inhaled short-acting bronchodilator treatment could not be controlled before CT scanning; therefore, PFT parameters before and after inhalation of the short-acting bronchodilator were compared using the air-trapping lung volume percentage.

Concordance analysis was used to assess the relationship between PFT parameters and quantitative CT assessment parameters. The concordance index between two parameters, standard error, and 95% confidence interval were calculated using the bootstrapping method^39^. A correlation matrix was generated with Pearson’s correlation analysis for overall assessment of the relationship of air-trapping lung volume percentage on chest CT with airway obstruction parameters such as tidal breathing ratio (t_PTEF_/t_E_) on PFT and ventilation inhomogeneity such as LCI, MR1, and MR2.

The correlation matrix, the partial correlation scatter plot of model 2, and the concordance analysis were conducted in the R statistical package (R version 3.2.5.; Institute for Statistics and Mathematics, Vienna, Austria; www.R-project.org). All other analyses were performed using SPSS version 23 (IBM Corp., Armonk, NY, USA). Any *p* value < 0.05 was considered statistically significant.

## Electronic supplementary material

Supplementary Information
