# Supplementary material for: Lung Clearance Index and Quantitative Computed Tomography of Post-Infectious Bronchiolitis Obliterans in Infants
Source: Sci Rep. 2017 Nov 9;7:15128. doi: 10.1038/s41598-017-15330-8 (PMC5680196; doi:10.1038/s41598-017-15330-8)
Supplement: Supplementary file 1 — Supplementary Information [file 41598_2017_15330_MOESM1_ESM.docx]

**Lung Clearance Index and Quantitative Computed Tomography of Post-Infectious Bronchiolitis Obliterans in Infants**

**Yoon Hee Kim, Hyun Joo Shin, In Suk Sol, Soo Yeon Kim, Jong Deok Kim, Haesung Yoon, Kyung Won Kim, Myung-Joon Kim, Mi-Jung Lee, and Myung Hyun Sohn**

**Online Data Supplement**

**Methods**

**Chest computed tomography (CT)**

Children randomly underwent chest CT scan using one of five CT scanners from two vendors according to the clinical demand. (Somatom Definition AS+, Sensation 64, and Somatom Definition Flash were from Siemens Medical Solutions, Erlangen, Germany; Discovery CT750 HD and Revolution CT were from GE Healthcare, WI, USA.) Tube voltages were 80 or 100 kVp, depending on the child’s weight, and automatic dose modulation was applied. Slice thickness was 1 or 2 mm, depending on the scanner and examination timing. Subjects were in supine position under sedation. Sedation with orally administered chloral hydrate (60−100 mg/kg) was performed by pediatricians or anesthetists without the need for intubation or ventilation. Subjects were in spontaneous free-breathing status; thus, images of deep inspiration or expiration, which are used for precise evaluation of air-trapping in small airway disease of adults and adolescents, could not be acquired. An inhaled short-acting bronchodilator treatment could not be controlled before CT scanning.

**Infant Pulmonary Function Tests (iPFT)**

Tidal breathing flow-volume loops (TBFVL) were obtained during quiet tidal breathing at least 30 seconds after initial mask placement. Recordings were analyzed if > 30 consecutive regular breaths of tidal breathing were recorded during a 90-second epoch and according to international guidelines ^1^. Mean tidal breathing measures were calculated using Wbreath v3.2.0 software (Ndd Medizintechnik AG, Zurich, Switzerland).

The MBW test was conducted with 4% SF_6_ as a tracer gas using an ultrasonic flow meter (Spiroson, Ecomedics AG, Durnten, Switzerland) with acquisition and analysis software (Wbreath v3.2.0). The tests were conducted according to the ERS/ATS recommendations for MBW measurements ^2^. The washout period began after a ten-breath equilibrium period at the end of the tracer gas wash-in. Washout continued until the tracer gas was eliminated from the lungs. The outcome parameters included: functional residual capacity (FRC), LCI (cumulative expired volume/FRC), moment ratio 1 (MR1; M_1_/M_0_), and moment ratio 2 (MR2; M_2_/M_0_), which were determined by plotting the normalized end-tidal SF_6_ concentration against the number of lung turnovers required to reach 1/40^th^ of the starting SF_6_ concentration ^2^. The recordings were defined as acceptable for analysis if they occurred during quiet sleep, with no sighs within 10 breaths of the wash-in plateau or 10 breaths after the SF_6_ concentration returned to baseline levels. The process was repeated to obtain three successful recordings. If the subject woke, testing was restarted after a return to sleep. The recordings were analyzed using Wbreath v3.2.0 software, including the use of the optimized temperature and dead space correction ^3^. Flow and volume were converted to body temperature and pressure under saturated conditions. Reported values represent the mean FRC and LCI for three complete tests. If the mean was within 10% of the lower value, the reported value represents the mean of two tests.

**References**

1. Bates, J. H., Schmalisch, G., Filbrun, D. & Stocks, J. Tidal breath analysis for infant pulmonary function testing. ERS/ATS Task Force on Standards for Infant Respiratory Function Testing. European Respiratory Society/American Thoracic Society. *Eur Respir J* **16**, 1180-1192 (2000).

2. Robinson, P. D. *et al.* Consensus statement for inert gas washout measurement using multiple- and single- breath tests. *Eur Respir J* **41**, 507-522 (2013).

3. Latzin, P. *et al.* Optimized temperature and deadspace correction improve analysis of multiple breath washout measurements by ultrasonic flowmeter in infants. *Pediatr Pulmonol* **42**, 888-897 (2007).
